# The osteoporosis–fracture paradox in older Taiwanese men: lower osteoporosis prevalence but comparable fragility fracture burden

**DOI:** 10.1007/s11657-026-01782-x

**Published:** 2026-10-31

**Authors:** Yi-Yen Lee, Yi-Chieh Huang, Sheng-Wen Kao, Yi-Chou Chen, Ming-Te Cheng

**Affiliations:** 1 Sinwu Branch, Taoyuan General Hospital, Ministry of Health and Welfare, Taoyuan City, Taiwan, ROC; 2 Department of Orthopaedic Surgery, Taoyuan General Hospital, Ministry of Health and Welfare, Taoyuan City, Taiwan, ROC; 3 National Defense Medical Center, Graduate Institute of Medical Sciences, Taipei City, Taiwan, ROC; 4 Department of Biomedical Engineering, Chung Yuan Christian University, Taoyuan City, Taiwan, ROC; 5 Department of Medical Science, National Tsing Hua University, Hsinchu, Taiwan, ROC

**Keywords:** Osteoporosis men, Fragility fracture, Dual-energy X-ray absorptiometry, Bone mineral density, Epidemiology

## Abstract

***Summary*:**

Despite a substantially lower prevalence of osteoporosis than age-matched women, older men experienced a relatively comparable burden of fragility fractures, demonstrating a marked dissociation between osteoporosis prevalence and fracture burden.

**Purpose:**

Although osteoporosis is less common in men than in women, male fragility fractures carry substantial morbidity and post-fracture mortality. Whether the lower prevalence of osteoporosis translates into a proportionally lower fracture burden remains unclear. We compared the prevalence of osteoporosis and fragility fractures between age-matched Taiwanese men and women.

**Methods:**

This retrospective cohort study evaluated 2093 men undergoing clinical dual-energy X-ray absorptiometry (DXA) between 2021 and 2023, of whom 2031 met eligibility criteria and were compared with 2031 age-matched women selected via 1:1 nearest-neighbor matching. Osteoporosis was defined as a T-score ≤−2.5 at the lumbar spine, femoral neck and/or total hip. Vertebral and hip fractures were identified from radiographs and electronic medical records. Multivariable logistic regression identified independent predictors of fracture.

**Results:**

The prevalence of osteoporosis in men was approximately half that in women (32.4% vs. 51.4%; *P* < 0.001). Conversely, vertebral fracture prevalence was comparable between the sexes (22.7% vs. 22.8%; *P* = 0.940), and hip fracture prevalence did not differ significantly between the sexes (6.1% vs. 5.0%; *P* = 0.150). In sensitivity analyses, men had a significantly higher prevalence of moderate-to-severe (Genant grade ≥2) vertebral fractures (16.9% vs. 12.3%; *P* < 0.001). After adjustment for age, body mass index, and osteoporosis, male sex remained independently associated with both vertebral and hip fractures.

**Conclusions:**

Older Taiwanese men exhibited a substantially lower prevalence of osteoporosis than age-matched women but experienced a relatively comparable burden of fragility fractures. These findings demonstrate a marked dissociation between osteoporosis prevalence and fragility fracture burden in older men and reinforce the importance of comprehensive fracture risk assessment integrating BMD with clinical risk factors.

## Introduction

Osteoporosis is a major public health challenge affecting more than 200 million people worldwide and is associated with substantial morbidity, mortality, loss of independence, and healthcare expenditure. Although historically regarded as a disease predominantly affecting postmenopausal women, osteoporosis in men is increasingly recognized as an important public health problem [1–5]. Approximately one in five men aged over 50 years will sustain an osteoporotic fracture during their lifetime, and men account for 25–40% of all fragility fractures despite having a substantially lower prevalence of osteoporosis than women [1–5]. Importantly, men experience higher mortality, greater disability, and poorer functional recovery following hip fracture than women, underscoring the considerable clinical burden of male osteoporosis [1–5].

The lower prevalence of osteoporosis in men is largely attributable to biological differences in skeletal development and ageing. Men generally achieve higher peak bone mass, larger skeletal size, and experience a slower rate of age-related bone loss because they do not undergo the abrupt hormonal changes associated with menopause [3–5]. Nevertheless, osteoporosis in men remains markedly underdiagnosed and undertreated, with many patients not diagnosed until after sustaining a fragility fracture [3–5]. Although anti-osteoporotic therapies effectively reduce vertebral and non-vertebral fractures in men [6], treatment rates remain low. Consequently, current international guidelines recommend integrating bone mineral density (BMD) with clinical risk factors and advocate routine DXA screening for men aged 70 years or older, or younger men with major clinical risk factors [7–10]. These recommendations have recently been reinforced by the Asia-Pacific consensus [11], and the REMBO trial, which demonstrated that systematic remote bone health services significantly increased osteoporosis detection and treatment initiation among high-risk men [12].

Despite these advances, an important epidemiological paradox remains. Large population-based studies consistently demonstrate that men have a substantially lower prevalence of osteoporosis than women, yet the between-sex difference in fragility fractures is considerably less pronounced. The China Osteoporosis Prevalence Study [13], a systematic review of Asia-Pacific populations [14], recent Korean nationwide data [15], and the Danish Nationwide Osteoporosis Cohort Trials Environment (NOCTE) [16] have consistently demonstrated a relatively low prevalence of osteoporosis in men, while population-based evidence has indicated a substantial burden of fragility fractures. Similarly, our previous Taiwanese study demonstrated a considerable burden of osteoporosis and fragility fractures among older women, emphasizing the substantial public health impact of skeletal fragility within Asian populations [17]. Collectively, these findings suggest that osteoporosis prevalence alone does not fully characterize fracture burden in older men.

Beyond bone density, fracture susceptibility cannot be fully explained by areal BMD alone. Prospective data from the Osteoporotic Fractures in Men (MrOS) study demonstrated that proximal femoral BMD strongly predicts hip and nonvertebral fractures in older men [18]. Alarkawi et al. further showed that lumbar spine BMD contributes incremental fracture prediction beyond hip BMD [19], while Leslie et al. demonstrated that regional variation in skeletal density provides clinically meaningful fracture information beyond conventional osteoporosis classification [20]. Consistent with these observations, our recent study of more than 4000 Taiwanese postmenopausal women demonstrated that spine–hip BMD discordance was associated with substantially different fracture burdens despite similar hip BMD [21]. In addition, pooled analyses from the FNIH-ASBMR-SABRE collaboration confirmed that fracture risk increases continuously across the spectrum of baseline BMD rather than only below the osteoporosis threshold [22]. Together, these observations suggest that fracture susceptibility reflects the combined influence of bone density, regional skeletal variation, and other determinants of skeletal fragility rather than the presence or absence of osteoporosis alone.

Nevertheless, relatively few studies have directly compared osteoporosis prevalence and fragility fracture burden between age-matched men and women examined using identical DXA protocols within the same healthcare system. Most available evidence is derived from national surveys or community-based cohorts with substantial differences in age distribution, ethnicity, referral patterns, and imaging methodology, limiting direct comparisons between the sexes. Whether the substantially lower prevalence of osteoporosis in men translates into a proportionally lower burden of fragility fractures therefore remains uncertain.

Therefore, we conducted an age-matched comparison of osteoporosis prevalence and fragility fracture burden between men and women undergoing routine clinical DXA examinations in a large Taiwanese hospital-based cohort. Osteoporosis was defined according to current international diagnostic criteria using lumbar spine, femoral neck, and total hip DXA measurements. We further evaluated the independent associations of sex, age, body mass index, and osteoporosis with vertebral and hip fractures using multivariable logistic regression. We hypothesized that, despite a substantially lower prevalence of osteoporosis, older men would exhibit a comparable burden of fragility fractures compared with age-matched women.

## Materials and methods

### Study design and participants

**Fig. 1:**
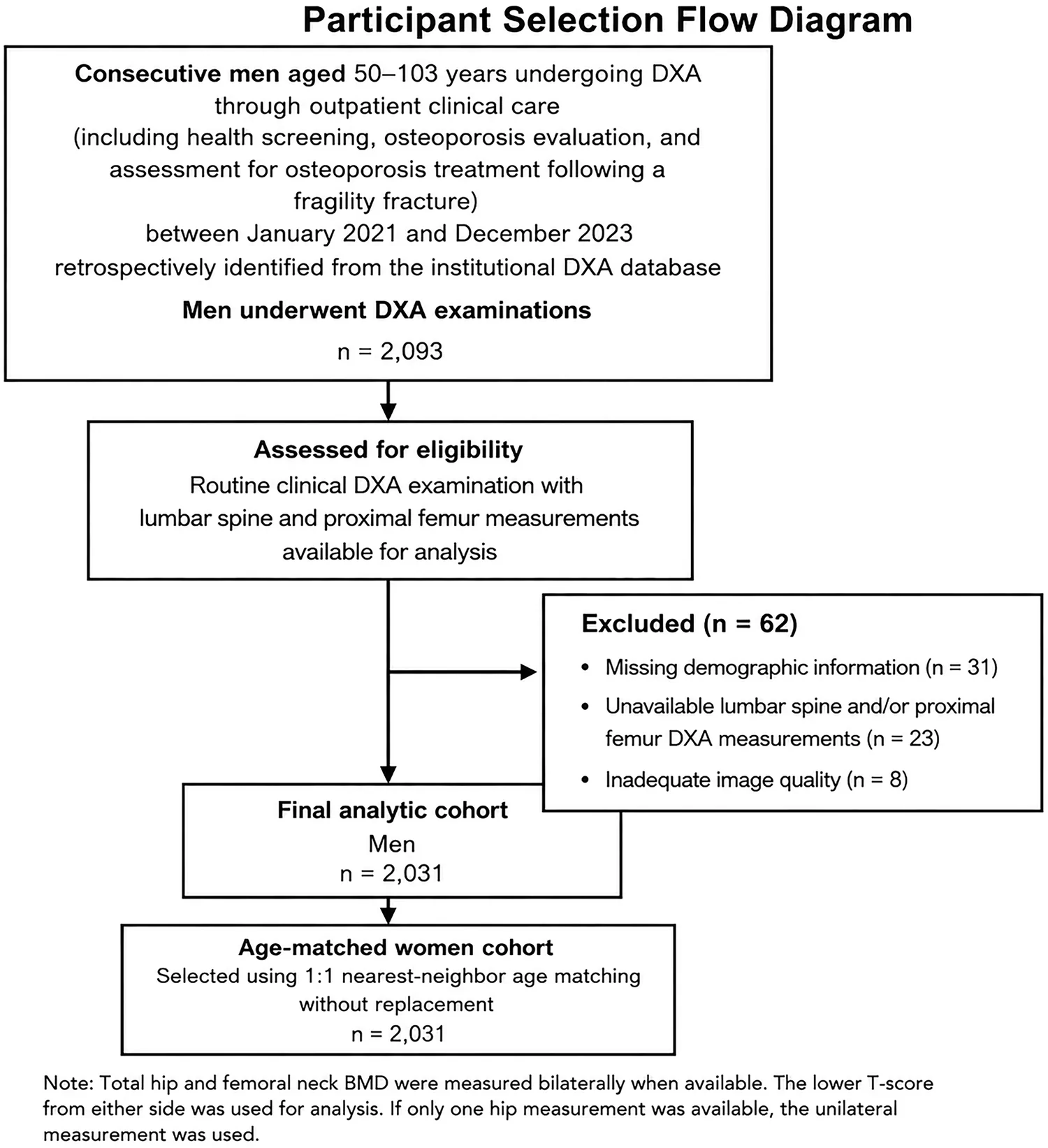
Participant selection flow diagram. Flow diagram illustrating participant selection for the study. Consecutive men aged 50–103 years undergoing DXA through outpatient clinic care, including health screening, osteoporosis evaluation, and assessment for osteoporosis treatment following a fragility fracture, between January 2021 and December 2023 were retrospectively identified from the institutional DXA database. Participants with missing demographic information, unavailable lumbar spine and/or proximal femur DXA measurements, or inadequate image quality were excluded. The final analytic cohort comprised 2031 men. An age-matched female cohort of 2031 women was subsequently selected using 1:1 nearest-neighbor age matching without replacement for comparative analyses

This retrospective age-matched cohort study was conducted at Taoyuan General Hospital, Ministry of Health and Welfare, Taiwan. Consecutive men aged 50–103 years undergoing DXA through outpatient clinic care between January 2021 and December 2023 were retrospectively identified from the institutional DXA database. DXA examinations were performed for health screening, osteoporosis evaluation, or assessment for osteoporosis treatment following a fragility fracture. The men included in this study underwent a bone-health evaluation protocol that included lateral thoracolumbar radiography for assessment of prevalent vertebral fractures. During the study period, 2093 men underwent routine DXA examinations. Participants were excluded because of missing demographic information (*n* = 31), unavailable lumbar spine and/or proximal femur DXA measurements (*n* = 23), or inadequate image quality precluding analysis (*n* = 8). After application of these eligibility criteria and exclusions, the final analytic cohort comprised 2031 men. Nearest-neighbor matching was subsequently performed using this final eligible male cohort. Women were selected from a previously established institutional DXA database using 1:1 nearest-neighbor age matching without replacement, with each man matched to the woman with the closest age, resulting in an age-matched female cohort of 2,031 participants. The participant selection process is summarized in Fig. 1. The study protocol was approved by the Institutional Review Board of Taoyuan General Hospital, Ministry of Health and Welfare, Taiwan (Approval No. TYGH114057). Owing to the retrospective study design and the use of de-identified clinical data, the requirement for written informed consent was waived.

### Clinical data collection

Demographic and anthropometric characteristics, including age, height, weight, and body mass index (BMI), were extracted from the electronic medical records. Body mass index was calculated as weight in kilograms divided by height in meters squared (kg/m²).

Fragility fractures were identified retrospectively through a comprehensive review of electronic medical records, radiology reports, and all available radiographic images obtained during routine clinical care. No fixed look-back interval was prespecified; historical records and imaging studies were reviewed as far back as documentation was available in the institutional system for each participant. Hip fractures were defined as low-energy femoral neck or intertrochanteric fractures resulting from a fall from standing height or less, confirmed by pelvic radiographs and corresponding clinical documentation. Vertebral fractures were assessed using available lateral thoracolumbar radiographs obtained within the routine clinical evaluation with a Toshiba MRAD-A50S digital radiography system and graded according to the Genant semi-quantitative method [23]. Available imaging studies and medical records were reviewed to distinguish fragility fractures from fractures associated with major trauma. Fractures with a documented history of major trauma were excluded from the fragility-fracture classification. All participants had lateral spine radiographs available for vertebral fracture assessment; however, radiographic coverage varied according to routine clinical practice, ranging from approximately T10–L4 to T4–L4. Vertebral fracture assessment (VFA) by DXA was not used. The primary outcome of “any fragility fracture” was defined as the presence of at least one vertebral and/or hip fracture. For vertebral fracture assessment, the available lateral thoracolumbar radiograph closest to the index DXA examination was evaluated. All fragility fractures, including vertebral and hip fractures, were independently reviewed by two board-certified orthopedic surgeons who were blinded to the DXA results. Discrepancies in fracture identification or grading were resolved by consensus, and interobserver agreement for the ordinal Genant grades was evaluated using the weighted Cohen’s kappa statistic. Fractures evaluated in this study were prevalent fractures identified retrospectively from available radiographs and electronic medical records rather than incident fractures prospectively ascertained after DXA. As a sensitivity analysis, moderate-to-severe vertebral fractures were defined as Genant grade ≥2 [23]. Their prevalence was compared between men and women, and independent associations with Genant grade ≥2 vertebral fractures were evaluated using the same multivariable logistic regression model as the primary analysis.

### Bone mineral density measurement

Bone mineral density (BMD) of the lumbar spine (L1–L4) and bilateral proximal femora was measured using dual-energy X-ray absorptiometry (DXA) with a Lunar Prodigy densitometer (GE Healthcare, Fairfield, CT, USA). In accordance with the 2023 ISCD Official Positions [9], total hip T-scores were generated using the NHANES III young-adult White female reference database, whereas lumbar spine T-scores were generated using the manufacturer-embedded GE Lunar young-adult White female reference database.

Osteoporosis was defined as a T-score ≤−2.5 at the lumbar spine, femoral neck, and/or total hip, in accordance with the World Health Organization diagnostic threshold and the Official Positions of the International Society for Clinical Densitometry [9, 24]. Participants not meeting the diagnostic threshold for osteoporosis were classified as having osteopenia or normal bone mineral density according to WHO criteria. One-third radius DXA was not routinely performed as part of the institutional DXA protocol during the study period and was therefore not included in the diagnostic definition of osteoporosis.

Vertebrae affected by structural abnormalities, previous spinal surgery, vertebral fractures, degenerative changes, or focal artifacts were excluded in accordance with ISCD Official Positions [9]. The lumbar spine T-score was calculated using the remaining evaluable vertebrae. Total hip and femoral neck BMD were measured bilaterally. The lower total hip and femoral neck T-score from either side was used. If only one hip measurement was available, the unilateral total hip and femoral neck T-score was used.

### Statistical analysis

The primary objectives were to compare the prevalence of osteoporosis and fragility fractures between age-matched men and women and to identify factors independently associated with fracture outcomes.

Continuous variables are presented as mean ± standard deviation (SD), and categorical variables as number (percentage). Between-group comparisons were performed using the independent-samples *t* test for continuous variables and the Pearson chi-square test for categorical variables.

Age-specific prevalence of osteoporosis and fragility fractures was summarized according to predefined age categories (50–59, 60–69, 70–79, and ≥80 years). Multivariable logistic regression analyses were performed to identify factors independently associated with fracture outcomes. Three primary regression models were constructed. Model 1 evaluated any fragility fracture as the dependent variable and included sex, age, and body mass index (BMI) as covariates. Model 2 evaluated vertebral fracture, and Model 3 evaluated hip fracture; both models included sex, age, BMI, and osteoporosis as independent variables. Results are presented as adjusted odds ratios (ORs) with 95% confidence intervals (CIs).

As a sensitivity analysis, the same multivariable logistic regression model used for vertebral fractures was repeated after restricting the outcome to moderate-to-severe vertebral fractures (Genant grade ≥2). In addition, an exploratory interaction analysis was performed to evaluate whether the associations between osteoporosis and fracture outcomes differed by sex. Statistical analyses were performed using Python (version 3.10) with the statsmodels and SciPy libraries. All statistical tests were two-sided, and a *P* value < 0.05 was considered statistically significant.

## Results

### Study population

A total of 2093 men underwent DXA in the outpatient setting for health screening, osteoporosis evaluation, or assessment for osteoporosis treatment following a fragility fracture between January 2021 and December 2023. After excluding participants with missing demographic information (*n* = 31), unavailable lumbar spine and/or proximal femur DXA measurements (*n* = 23), and inadequate image quality (*n* = 8), 2031 men were included in the final analysis. A 1:1 age-matched female cohort comprising 2031 women was selected from the institutional DXA database using nearest-neighbor matching without replacement (Fig. 1).

### Baseline characteristics

Baseline characteristics of the age-matched cohorts are summarized in Table 1. As expected, the mean age was comparable between men and women because of age matching. Men were significantly taller, heavier, and had a higher body mass index (BMI) than women (all *P* < 0.001). Bone mineral density was consistently higher in men across all measured skeletal sites, with significantly higher lumbar spine, femoral neck, and total hip T-scores than in women (all *P* < 0.001).

**Table 1:** Baseline characteristics of age-matched male and female participants

Characteristic	Male (*n* = 2031)	Female (*n* = 2031)	*P* value
Age, years	66.2 ± 10.9	66.3 ± 10.8	0.818
Height, cm	167.5 ± 6.8	155.0 ± 6.3	<0.001
Weight, kg	69.8 ± 12.0	57.3 ± 9.6	<0.001
Body mass index, kg/m²	24.8 ± 3.6	23.9 ± 3.7	<0.001
Lumbar spine T-score	−0.7 ± 1.6	−1.7 ± 1.5	<0.001
Total hip T-score	−1.2 ± 1.2	−1.6 ± 1.2	<0.001
Femoral neck T-score	−1.8 ± 1.1	−2.3 ± 1.0	<0.001
Vertebral fracture, n (%)	461 (22.7)	463 (22.8)	0.940
Hip fracture, n (%)	123 (6.1)	102 (5.0)	0.150

Data are presented as mean ± SD or number (percentage). Total hip and femoral neck T-scores represent the lower value of bilateral measurements when available; when only one hip measurement was available, the unilateral measurement was used. *P* values were calculated using the independent-samples t test for continuous variables and the chi-square test for categorical variables

Despite these more favorable anthropometric characteristics and significantly higher lumbar spine, femoral neck, and total hip BMD, men did not exhibit a proportionally lower burden of fragility fractures than women. Vertebral and hip fracture prevalence were comparable between the sexes (Table 1). Interobserver agreement for Genant vertebral fracture grading was substantial (weighted Cohen’s κ = 0.80). These findings indicate that the substantially lower prevalence of osteoporosis in men was not accompanied by a correspondingly lower prevalence of fragility fractures.

### Osteoporosis prevalence and age-specific distribution

**Fig. 2:**
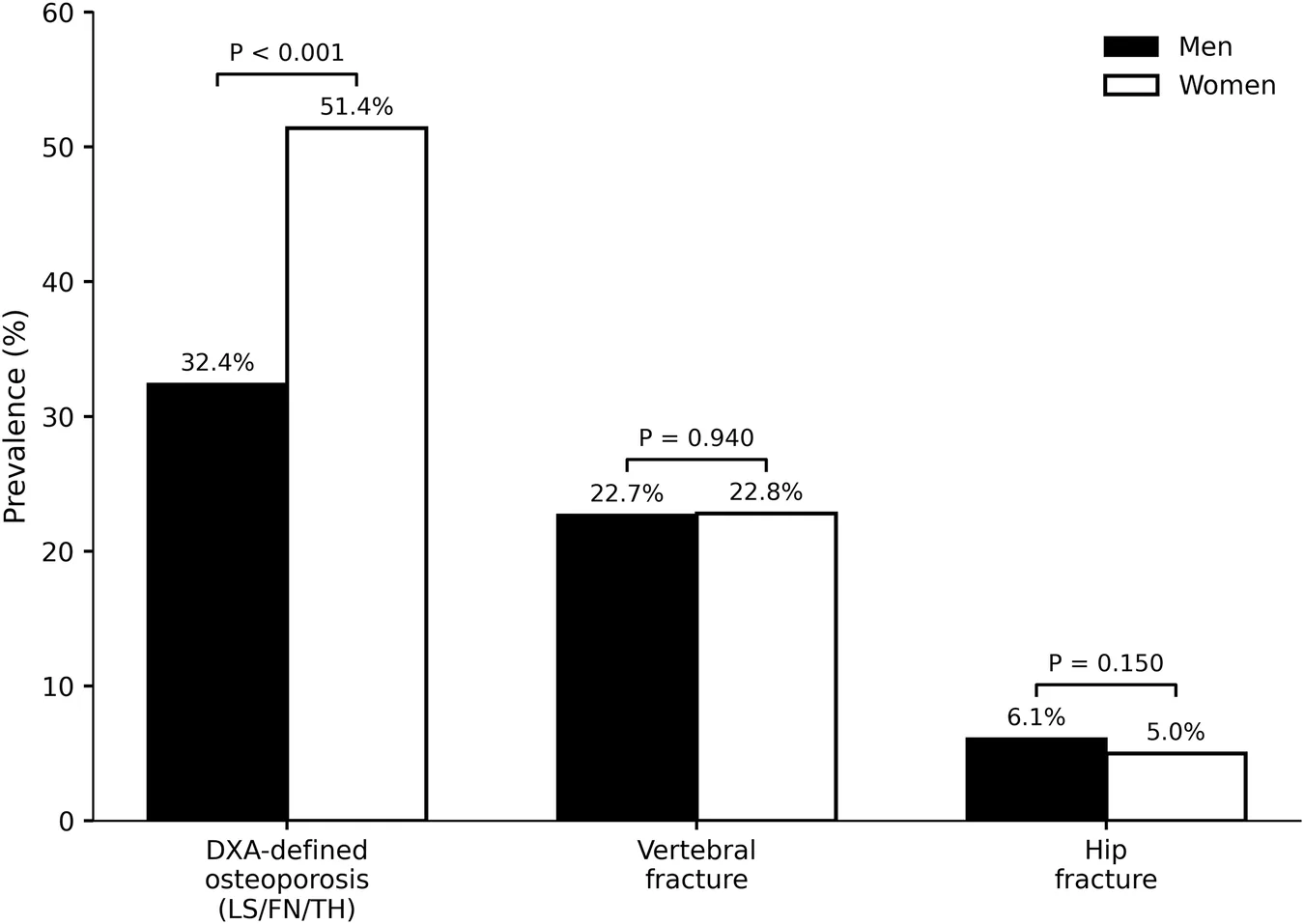
Comparison of osteoporosis and fragility fracture prevalence between age-matched men and women. Prevalence of osteoporosis, vertebral fracture, and hip fracture among 2031 age-matched men and 2031 women. Osteoporosis was defined as a T-score ≤−2.5 at the lumbar spine, femoral neck, and/or total hip. Men had a significantly lower prevalence of osteoporosis than women (32.4% vs. 51.4%, *P* < 0.001), whereas the prevalence of vertebral fracture (22.7% vs. 22.8%, *P* = 0.940) and hip fracture (6.1% vs. 5.0%, *P* = 0.150) did not differ significantly between sexes. Bars represent percentages. *P* values were calculated using the chi-square test

The prevalence of osteoporosis and fragility fractures is summarized in Table 2 and illustrated in Fig. 2. Overall, osteoporosis was identified in 32.4% of men compared with 51.4% of age-matched women (*P* < 0.001). Lumbar spine, femoral neck, and total hip osteoporosis were significantly less prevalent in men than in women (all *P* < 0.001). In contrast, the prevalence of both vertebral and hip fractures was comparable between the sexes (Table 2).

**Table 2:** Comparison of osteoporosis prevalence and fragility fractures between age-matched men and women

Variable	Male (%) (*n* = 2031)	Female (%) (*n* = 2031)	*P* value
Overall osteoporosis^a^	659 (32.4%)	1043 (51.4%)	<0.001
Lumbar spine osteoporosis	265 (13.0)	642 (31.6)	<0.001
Total hip osteoporosis	285 (14.0)	473 (23.3)	<0.001
Femoral neck osteoporosis	603 (29.7%)	877 (43.2%)	<0.001
Osteopenia	998 (49.1%)	835 (41.1%)	<0.001
Normal bone mineral density	374 (18.4%)	153 (7.5%)	<0.001
Vertebral fracture	461 (22.7)	463 (22.8)	0.940
Hip fracture	123 (6.1)	102 (5.0)	0.150

^a^Osteoporosis was defined as a T-score ≤−2.5 at the lumbar spine, femoral neck and/or total hip according to the World Health Organization diagnostic threshold and the International Society for Clinical Densitometry (ISCD) Official Positions. Data are presented as number (percentage). *P* values were calculated using the chi-square test

Age-stratified analyses demonstrated progressive increases in the prevalence of osteoporosis and fragility fractures with advancing age in both sexes (Table 3). Across all age groups, men consistently exhibited lower prevalence of osteoporosis than age-matched women. In contrast, vertebral and hip fracture prevalence increased progressively with age in both sexes, and the between-sex differences remained substantially smaller than those observed for osteoporosis across all age groups.

**Table 3:** Age-stratified prevalence of osteoporosis and fragility fractures

Age group	Male osteoporosis *n* (%)	Female osteoporosis *n* (%)	Male VF (%)	Female VF (%)	Male Hip Fx (%)	Female Hip Fx (%)
50–59	110 (16.6)	174 (26.2)	30 (4.5)	26 (3.9)	13 (2.0)	5 (0.8)
60–69	183 (28.1)	337 (51.7)	97 (14.9)	100 (15.3)	25 (3.8)	25 (3.8)
70–79	203 (43.7)	325 (69.9)	172 (37.0)	174 (37.4)	37 (8.0)	25 (5.4)
≥80	163 (65.2)	207 (82.8)	162 (64.8)	163 (65.2)	48 (19.2)	47 (18.8)
Overall	659 (32.4)	1,043 (51.4)	461 (22.7)	463 (22.8)	123 (6.1)	102 (5.0)

Data are presented as number (percentage). Osteoporosis was defined as a T-score ≤−2.5 at the lumbar spine, femoral neck and/or total hip

*VF* vertebral fracture, *Hip Fx* hip fracture

As a sensitivity analysis, moderate-to-severe vertebral fractures (Genant grade ≥2) were evaluated separately. Men exhibited a significantly higher prevalence of Genant grade ≥2 vertebral fractures than age-matched women (16.9% vs. 12.3%; *P* < 0.001; Supplementary Table S2). Multivariable logistic regression using the same covariates as the primary vertebral fracture model demonstrated that male sex, advancing age, body mass index, and osteoporosis remained independently associated with Genant grade ≥2 vertebral fractures (Supplementary Table S3).

### Multivariable logistic regression analyses

Multivariable logistic regression analyses are presented in Table 4. In Model 1, advancing age was independently associated with any fragility fracture, whereas neither male sex nor body mass index (BMI) was independently associated with the composite fracture outcome after adjustment. In Models 2 and 3, advancing age and osteoporosis were independently associated with both vertebral and hip fractures. After adjustment for age, BMI, and osteoporosis, male sex remained independently associated with vertebral and hip fractures, whereas BMI was independently associated with vertebral fracture but not hip fracture. Results are presented as adjusted odds ratios (ORs) with 95% confidence intervals in Table 4.

**Table 4:** Multivariable logistic regression analyses of factors associated with fragility fractures

Predictor	Model 1 Any fragility fracture^a^		Model 2 Vertebral fracture^b^		Model 3 Hip fracture^b^	
	Adjusted OR (95% CI)	*P* value	Adjusted OR (95% CI)	*P* value	Adjusted OR (95% CI)	*P* value
Male sex	1.11 (0.94–1.31)	0.205	1.21 (1.01–1.44)	0.036	1.58 (1.19–2.11)	0.002
Age (per year)	1.13 (1.12–1.14)	<0.001	1.12 (1.10–1.13)	<0.001	1.07 (1.05–1.08)	<0.001
BMI (per kg/m²)	1.01 (0.98–1.03)	0.642	1.06 (1.03–1.08)	<0.001	0.97 (0.93–1.01)	0.091
Osteoporosis	—	—	2.51 (2.07–3.03)	<0.001	3.43 (2.39–4.92)	<0.001

Data are presented as adjusted odds ratios (ORs) with 95% confidence intervals (CIs)

^a^Model 1 was adjusted for sex, age, and body mass index (BMI).

^b^Models 2 and 3 were adjusted for sex, age, body mass index (BMI), and overall osteoporosis. Osteoporosis was defined as a T-score ≤−2.5 at the lumbar spine, femoral neck, and/or total hip. Any fragility fracture was defined as vertebral fracture and/or hip fracture. A two-sided *P* value < 0.05 was considered statistically significant

Exploratory interaction analyses demonstrated a significant sex × osteoporosis interaction for vertebral fracture but not hip fracture (Supplementary Table S1), suggesting that the relationship between osteoporosis and vertebral fracture differs between men and women, whereas the association with hip fracture appears similar across sexes.

## Discussion

### Principal findings

In this age-matched DXA cohort, we identified three principal findings. First, men exhibited a substantially lower prevalence of osteoporosis than women despite an identical age distribution. Second, as illustrated in Fig. 2, the differences in vertebral and hip fracture prevalence between the sexes were considerably smaller than those in osteoporosis prevalence, revealing a marked dissociation between osteoporosis and fragility fracture burden in men. Third, after adjustment for age, body mass index (BMI), and osteoporosis, male sex remained independently associated with both vertebral and hip fractures. Collectively, these findings demonstrate a marked dissociation between osteoporosis prevalence and fragility fracture burden in older men and reinforce the need for fracture risk assessment strategies that integrate BMD with broader clinical risk factors.

### Comparison with previous studies

Our findings are consistent with contemporary evidence demonstrating that osteoporosis in men remains an important but underrecognized public health problem. Approximately one in five men aged over 50 years will sustain an osteoporotic fracture during their lifetime, and men account for 25–40% of all fragility fractures despite a substantially lower prevalence of osteoporosis than women [1–5]. Moreover, men experience higher mortality and poorer functional recovery following hip fracture, underscoring the disproportionate clinical consequences of skeletal fragility in this population [1–5]. Our study extends previous epidemiological observations by directly comparing age-matched men and women examined using identical DXA protocols within the same healthcare system. Consistent with the China Osteoporosis Prevalence Study [13], the Asia-Pacific systematic review [14], and the Danish Nationwide Osteoporosis Cohort Trials Environment (NOCTE) [16], we observed a substantially lower prevalence of osteoporosis in men despite a relatively comparable burden of fragility fractures. Together with our previous Taiwanese study in women [17], these findings reinforce that osteoporosis remains underrecognized in older men and support greater awareness of fracture risk across both sexes.

### The osteoporosis–fracture paradox in men

The principal contribution of this study is the demonstration of an osteoporosis–fracture paradox in older men. Although the prevalence of osteoporosis in men was substantially lower than that in age-matched women, vertebral and hip fracture prevalence differed far less between the sexes. This dissociation indicates that fracture burden is not directly proportional to the prevalence of osteoporosis and suggests that important determinants of skeletal fragility extend beyond conventional DXA-based classification. This interpretation is consistent with accumulating evidence that fracture susceptibility reflects multiple determinants rather than osteoporosis alone. The BHOF Clinician’s Guide and the National Bone Health Alliance position statement emphasize that fracture risk assessment should integrate BMD with clinical risk factors rather than relying solely on T-score thresholds [7, 8]. Large cohort studies, including the FNIH–ASBMR–SABRE [22] and MrOS [18], have likewise demonstrated that fracture risk increases continuously across the spectrum of BMD rather than being confined to individuals meeting the osteoporosis threshold. Our previous study further demonstrated that women with spine–hip BMD discordance experienced markedly different fracture burdens despite comparable hip BMD, supporting the concept that skeletal fragility reflects regional skeletal heterogeneity in addition to absolute bone density [21]. Together, these findings provide additional evidence that osteoporosis alone does not fully explain fracture susceptibility. Importantly, this observation persisted after expanding the diagnostic definition of osteoporosis to include the femoral neck in addition to the lumbar spine and total hip. Furthermore, when the analysis was restricted to moderate-to-severe vertebral fractures (Genant grade ≥2), men exhibited a significantly higher prevalence than age-matched women despite their substantially lower prevalence of osteoporosis, suggesting that clinically important vertebral fractures may be under-recognized in older men.

### Biological interpretation

Several mechanisms may contribute to the observed osteoporosis–fracture paradox. Although BMD is a major determinant of fracture risk, fracture susceptibility is also influenced by skeletal geometry, age-related changes in bone structure, falls, sarcopenia, and other clinical risk factors that are not captured by DXA alone [2, 22]. Degenerative changes may also influence lumbar spine BMD measurements in older adults, as osteophytes and other degenerative changes can artifactually elevate areal BMD and contribute to apparent spine–hip discordance. Although vertebrae with significant degenerative abnormalities were excluded from lumbar spine analysis according to ISCD recommendations, residual degenerative effects cannot be completely excluded. Consequently, individuals with similar DXA measurements may exhibit substantially different fracture susceptibility. Consistent with this concept, Alarkawi et al. demonstrated that lumbar spine BMD provides incremental fracture prediction beyond femoral neck BMD [19]. Similarly, our previous Taiwanese study demonstrated that spine–hip BMD discordance was associated with fracture burden beyond hip BMD alone [21]. Together, these findings suggest that fracture risk reflects skeletal and clinical factors rather than osteoporosis alone. Future prospective studies integrating DXA with advanced skeletal imaging and longitudinal fracture follow-up are warranted to better define the mechanisms underlying this osteoporosis–fracture paradox.

### Clinical implications

Our multivariable analyses demonstrated that advancing age, male sex, and osteoporosis remained independently associated with both vertebral and hip fractures. These findings are consistent with the well-established concept that T-score thresholds do not fully capture fracture risk and reinforce current recommendations that fracture risk assessment should integrate BMD with clinical risk factors, as incorporated in tools such as FRAX, rather than relying exclusively on osteoporosis [7–12]. Together with our previous work demonstrating clinically important skeletal heterogeneity in women [21], these findings suggest that fracture risk assessment may benefit from considering BMD together with other skeletal and clinical risk factors, rather than relying exclusively on T-score thresholds. Exploratory interaction analyses further suggest that the association between osteoporosis and vertebral fracture may differ by sex. Although exploratory, these findings warrant further investigation of sex-specific patterns of fracture susceptibility. From a clinical perspective, our findings support a more proactive approach to osteoporosis screening in older men. Despite a substantially lower prevalence of osteoporosis, men experienced a relatively comparable burden of vertebral and hip fractures. Because prevalent vertebral fractures strongly predict subsequent fractures, earlier identification of high-risk men represents an important opportunity for intervention. Together with the REMBO trial and the recent Asia-Pacific consensus [11, 12], our findings provide additional epidemiological evidence supporting expanded case finding and earlier evidence-based treatment in older men.

### Strengths and limitations

The principal strengths of this study include age-matched male and female cohorts derived from the same institution using identical DXA equipment and standardized acquisition protocols, minimizing variability related to BMD measurement. The use of a single DXA machine throughout the study ensured consistency in image acquisition and T-score determination. Direct comparison within a single healthcare system enabled robust evaluation of sex-specific differences. Sensitivity analyses restricted to Genant grade ≥2 vertebral fractures and exploratory interaction analyses further supported the robustness of the primary findings. Several limitations should be acknowledged. First, this was a retrospective single-center study, which may limit the generalizability of our findings to other populations and healthcare settings. In addition, men and women may have undergone DXA for different clinical indications. The male cohort included individuals undergoing health screening as well as those referred for osteoporosis evaluation or assessment for treatment following a fragility fracture. Although age matching ensured comparable age distributions and all participants were drawn from the same institution, referral bias cannot be excluded, as the male cohort may have been enriched for individuals with a higher baseline risk of osteoporosis or fracture than women. Second, important risk factors including smoking, alcohol consumption, hypogonadism, glucocorticoid exposure, falls, physical activity, and other secondary causes of osteoporosis were unavailable and therefore could not be included in the multivariable analyses. Third, although all participants had lateral spine radiographs available for vertebral fracture assessment, radiographic coverage was not uniform, with some examinations limited to the lower thoracic and lumbar spine. Therefore, upper thoracic vertebral fractures may have been underascertained, and the timing of radiographs relative to DXA was not standardized. In addition, because fracture history was ascertained from all available historical records without a prespecified look-back interval, the duration of available retrospective documentation may have varied among participants. Fourth, although all DXA examinations were performed using a single densitometer with standardized acquisition protocols, a dedicated retrospective review of all DXA scans was not performed specifically for this study; therefore, undetected acquisition or analysis errors cannot be completely excluded. Fifth, planar DXA does not capture volumetric density, bone geometry, cortical porosity, or trabecular microarchitecture, all of which contribute to skeletal fragility. Finally, the cross-sectional design precludes causal inference and longitudinal assessment of incident fracture risk.

## Conclusion

In conclusion, Taiwanese men exhibited a substantially lower prevalence of osteoporosis than age-matched women yet experienced a relatively comparable burden of vertebral and hip fragility fractures. These findings reinforce the importance of comprehensive fracture risk assessment integrating BMD with broader clinical risk factors in older men. Earlier recognition and treatment of osteoporosis in older men may therefore represent an important opportunity to reduce future fracture burden.

## Supplementary information

Supplementary Table S1 clean

Supplementary Table S2

Supplementary Table S3 clean

## Data Availability

The datasets generated and/or analyzed during the current study are not publicly available because of patient privacy and institutional restrictions, but are available from the corresponding author upon reasonable request and with permission from Taoyuan General Hospital.
