# Supplementary material for: The osteoporosis–fracture paradox in older Taiwanese men: lower osteoporosis prevalence but comparable fragility fracture burden
Source: Arch Osteoporos. 2026 Oct 31;21(1):156. doi: 10.1007/s11657-026-01782-x (PMC13723678; doi:10.1007/s11657-026-01782-x)
Supplement: Supplementary file 1 [file 11657_2026_1782_MOESM1_ESM.docx]

**Supplementary Table S1. Interaction between sex and osteoporosis for vertebral and hip fractures**

| Outcome | Predictor | Adjusted OR (95% CI) | P value |
| --- | --- | --- | --- |
| Vertebral fracture | Male sex | 0.89 (0.68–1.17) | 0.398 |
|  | Age (per year) | 1.12 (1.11–1.13) | <0.001 |
|  | BMI (per kg/m²) | 1.06 (1.04–1.09) | <0.001 |
|  | Osteoporosis | 1.94 (1.49–2.53) | <0.001 |
|  | Male sex × osteoporosis | 1.62 (1.14–2.31) | 0.008 |
| Hip fracture | Male sex | 1.63 (1.01–2.63) | 0.047 |
|  | Age (per year) | 1.07 (1.06–1.08) | <0.001 |
|  | BMI (per kg/m²) | 0.97 (0.93–1.01) | 0.152 |
|  | Osteoporosis | 2.95 (1.82–4.76) | <0.001 |
|  | Male sex × osteoporosis | 0.98 (0.53–1.79) | 0.942 |

Footnotes. Data are presented as adjusted odds ratios (ORs) with 95% confidence intervals (CIs). Models included sex, age, body mass index (BMI), osteoporosis, and the sex × osteoporosis interaction term. Osteoporosis was defined as a T-score ≤ −2.5 at the lumbar spine, femoral neck, and/or total hip. The male sex coefficient represents the association between sex and fracture among participants without osteoporosis, whereas the osteoporosis coefficient represents the association between osteoporosis and fracture among women. The interaction term tests whether the association between osteoporosis and fracture differs by sex. A two-sided P value < 0.05 was considered statistically significant.
