# Supplementary material for: The osteoporosis–fracture paradox in older Taiwanese men: lower osteoporosis prevalence but comparable fragility fracture burden
Source: Arch Osteoporos. 2026 Oct 31;21(1):156. doi: 10.1007/s11657-026-01782-x (PMC13723678; doi:10.1007/s11657-026-01782-x)
Supplement: Supplementary file 2 [file 11657_2026_1782_MOESM2_ESM.docx]

**Supplementary Table S2. Comparison of Moderate-to-severe Vertebral Fractures (Genant grade ≥2) between Age-matched Men and Women**

| Variable | Men (n=2,031) | Women (n=2,031) | P value |
| --- | --- | --- | --- |
| Genant grade ≥2 vertebral fracture | 344 (16.9%) | 250 (12.3%) | <0.001 |

**Footnote.** Values are presented as number (percentage). P values were calculated using the chi-square test. Moderate-to-severe vertebral fracture was defined as **Genant grade ≥2 (Genant grades 2 and 3).**
