# Supplementary material for: The osteoporosis–fracture paradox in older Taiwanese men: lower osteoporosis prevalence but comparable fragility fracture burden
Source: Arch Osteoporos. 2026 Oct 31;21(1):156. doi: 10.1007/s11657-026-01782-x (PMC13723678; doi:10.1007/s11657-026-01782-x)
Supplement: Supplementary file 3 [file 11657_2026_1782_MOESM3_ESM.docx]

**Supplementary Table S3. Multivariable logistic regression analysis for Genant grade ≥2 vertebral fracture**

| **Predictor** | **Adjusted OR (95% CI)** | **P value** |
| --- | --- | --- |
| Male sex | 2.05 (1.66–2.53) | <0.001 |
| Age (per year) | 1.11 (1.10–1.12) | <0.001 |
| BMI (per kg/m²) | 1.06 (1.03–1.09) | <0.001 |
| Osteoporosis | 2.84 (2.26–3.56) | <0.001 |

**Footnote.** Data are presented as adjusted odds ratios (ORs) with 95% confidence intervals (CIs). The outcome was **moderate-to-severe vertebral fracture (Genant grade ≥2).** The model included sex, age, body mass index (BMI), and osteoporosis. **Osteoporosis was defined as a T-score ≤ −2.5 at the lumbar spine, femoral neck** and/or **total hip.** The analysis included 2,031 men and 2,031 age-matched women. A two-sided **P value** < 0.05 was considered statistically significant.
